# Biochemical systems identification by a random drift particle swarm optimization approach

**DOI:** 10.1186/1471-2105-15-s6-s1

**Published:** 2014-05-16

**Authors:** Jun Sun, Vasile Palade, Yujie Cai, Wei Fang, Xiaojun Wu

**Affiliations:** 1 Key Laboratory of Advanced Process Control for Light Industry Ministry of Education, Jiangnan University, No. 1800, Lihu Avenue, Wuxi, Jiangsu 214122, China; 2 Faculty of Engineering and Computing, Coventry University, Priory Street, Coventry, CV1 5FB, UK; 3 Key Laboratory of Industrial Technology, School of Biotechnology, Jiangnan University, No. 1800, Lihu Avenue, Wuxi, Jiangsu 214122, China

## Abstract

**Background:**

Finding an efficient method to solve the parameter estimation problem (inverse problem) for nonlinear biochemical dynamical systems could help promote the functional understanding at the system level for signalling pathways. The problem is stated as a data-driven nonlinear regression problem, which is converted into a nonlinear programming problem with many nonlinear differential and algebraic constraints. Due to the typical ill conditioning and multimodality nature of the problem, it is in general difficult for gradient-based local optimization methods to obtain satisfactory solutions. To surmount this limitation, many stochastic optimization methods have been employed to find the global solution of the problem.

**Results:**

This paper presents an effective search strategy for a particle swarm optimization (PSO) algorithm that enhances the ability of the algorithm for estimating the parameters of complex dynamic biochemical pathways. The proposed algorithm is a new variant of random drift particle swarm optimization (RDPSO), which is used to solve the above mentioned inverse problem and compared with other well known stochastic optimization methods. Two case studies on estimating the parameters of two nonlinear biochemical dynamic models have been taken as benchmarks, under both the noise-free and noisy simulation data scenarios.

**Conclusions:**

The experimental results show that the novel variant of RDPSO algorithm is able to successfully solve the problem and obtain solutions of better quality than other global optimization methods used for finding the solution to the inverse problems in this study.

## Background

Evolutionary algorithms (EAs) have been widely used for data mining tasks in Bioinformatics and Computational Biology [1,2]. They are random search methods inspired by natural mechanisms existing in the biological world [1,2]. EAs originally comprised four types of paradigms, namely, genetic algorithms (GAs), genetic programming (GP), evolution strategies (ES), and evolutionary programming (EP), with GAs being the most popular one. Data analysis tools traditionally used in Bioinformatics were mainly based on statistical techniques, such as regression and estimation, and EAs played significant roles in handling large biological data sets in a robust and computationally efficient manner [2].

Currently, evolutionary computing techniques mostly comprise conventional EAs (GAs, GP, ES and EP), swarm intelligence algorithms, artificial immune systems, differential evolution, as the main representative classes of evolutionary computing approaches[3]. Swarm intelligence is a class of evolutionary computing techniques simulating natural systems composed of many individuals that coordinate one another using decentralized control and self-organization. Two most influential and classical examples of swarm intelligence approaches are particle swarm optimization (PSO) and ant colony optimization (ACO) algorithms, which have been widely used in many different fields [3-7]. Particularly, PSO algorithms have shown their effectiveness in data mining tasks in bioinformatics due to their performance in solving difficult optimisation tasks [8-10].

Biochemical modelling can be considered a generic data-driven regression problem on the given experimental data. The goal of biochemical modeling is to build the mathematical formulations that quantitatively describe the dynamical behaviour of biochemical processes. For example, metabolic reactions are formulated as rate laws and described as a system of differential equations, the kinetic parameters of which are identified from a set of experimental data. Finding the solution of the parameter estimation problem, thus, plays a key role in building a dynamic model for a biochemical process, which, in turn, can help understand the functionality of the signalling pathways at the system level [11,12].

Since solving the inverse problem in biochemical process modelling involves a task of nonlinear programming, many numerical optimization methods have been used to determine the parameters of biochemical models. These methods can be generally classified into two categories, namely, local optimization methods and global optimization methods [13]. The widely used local optimization tools for inverse problems are those based on gradient descent methods, the most popular being the Newton method [14]. This type of approaches, however, cannot be applied to non-smooth problems, since the objective functions of the problems are discontinuous or have discontinuous derivatives. Direct search methods, such as the Hooke-Jeeves method, the Needler-Mead simplex algorithm and the Downhill simplex algorithm, are also a kind of local optimization techniques that could be used to find a local minimum without the information from derivatives [13]. Normally, most local optimization approaches are used as single shooting methods. For each of them, the path of its optimization process leading to a final solution is determined by the initial conditions for the state variables. Therefore, the algorithm will lead to a wrong minimum, particularly if the initial conditions depend on model parameters. To overcome this shortcoming, one can adopt multiple shooting methods in which the time interval is partitioned and new initial conditions are used at the start of each time interval part [15]. The methods can offer the possibility to circumvent local optima by enlarging the parameter space during the optimization process.

The aforementioned local search methods are generally less efficient for the inverse problems of biochemical models, which are multimodal and high-dimensional. In order to solve these hard inverse problems efficiently, one can turn to global optimization methods, most of which incorporate stochastic search strategies to prevent the search process from being stuck into the local optimal or suboptimal solutions. The branch-and-bound approach is a global optimization method that converts the inverse problem into a convex optimization problem so that a global optimal solution can be obtained [16]. This method requires a finite search space that can be divided into smaller subspaces. A remarkable disadvantage is that it is applicable only if the lower and upper bounds of the objective function can be computed. Simulated annealing (SA) can be effectively used for parameter estimation from time-course biochemical data as shown in [17]. However, it has a slow convergence speed and high computational cost, and is not easy to be parallelized. Genetic algorithms (GAs) represent a widely used global search technique that could be employed to predict the parameters of dynamic models [18]. Nevertheless, GAs are always complained of slow convergence speed and high computation cost. The evolutionary strategy (ES) approach showed its ability to successfully solve inverse problems in a performance comparison made by Moles et al. [19] among a number of global optimization techniques on biochemical system identification problems. In contrast to SA, evolutionary algorithms, including ES and GAs, can be implemented as self-tuning methods and can be parallelizable, with the stochastic ranking evolutionary strategy (SRES) method being a very successful example [19-21]. Scatter search (SS) is known as a population-based random search approach that was proposed to identify the appropriate parameters for nonlinear dynamic biological systems [22,23]. As an evolutionary algorithm method, the SS method, as well as its hybrid with a local search step after the recombination operation, showed to be efficient in solving inverse problems. Particle swarm optimization (PSO), also a population-based optimization technique from swarm intelligence and evolutionary computation area, has demonstrated its better performance than GAs in solving inverse problems [24,25]. Hybrids of PSO with other methods have also shown their effectiveness in modelling biochemical dynamic systems [26-28]. However, PSO shows to be sensitive to the neighbourhood topology of the swarm, as commented in [29].

Other methods for parameter estimation include the Newton-flow analysis [30], the alternating regression technique [31], decoupling approaches [32,33], the collocation method [20,34], the decomposing method [35,36]. These approximation techniques, when incorporated into an optimization algorithm, can help reduce the number of objective function evaluations, which are very computationally expensive. Additionally, radial basis function neural networks [37] and a quantitative inference method [38] have also been employed to solve inverse problems in biochemical process modelling.

In all of the above cases, the optimization approach is used to minimize to the residual error of an inferred model against experimental data. Smaller error means that the model describes the dynamic behaviour of the biochemical system better and has more justification to be accepted as a valid mathematical representation of the system. Theoretically, the prediction error diminishes with the accuracy of the model increasing. This study focuses on developing an efficient optimization method for parameter estimation of a given dynamic biochemical model. However, since parameter estimation problems of complex dynamic systems (generally with many parameters and many differential equations) are high-dimensional, multimodal and more challenging to solve, but which allow to depict more complex biochemical processes, our goal in this study is to develop an efficient global optimization method for solving such inverse problems of complex biochemical dynamic systems.

After extensive and in-depth study, we selected the PSO algorithm as a candidate to be modified in order to achieve our goal of solving complex inverse problems. The reason why PSO attracted us is that PSO has many advantages, such as faster convergence speed, lower computational need, as well as being easily parallelizable and having fewer parameters to adjust. However, PSO has the following shortcomings. First of all, it was theoretically proven that the PSO is not a global convergent algorithm, even not a local convergent one, against the convergence criteria given in [39]. Practically, the algorithm is more prone to be trapped into local optimal or suboptimal points for a high-dimensional problem, due to the weakening of its global search ability during the mid and later stages of the search process. Next, PSO is widely known to be sensitive to its search parameters including upper limits of the velocity, and even to the "swarm topology", so that users may feel awkward when selecting the parameters and the topologies when using the algorithm [40]. Finally, the performance of PSO appears to be very sensitive to the setting of upper and lower bounds of the search scope [40]. If the global optimal solution is located near the boundary of the search scope, the algorithm may have little chance to catch it. We have found that these shortcomings are mainly attributed to the velocity update equation, which is the essence of the PSO algorithm, and where it seems to be much room for improvement so as to boost the global search ability of the PSO.

In this study, inspired by the free electron model in metal conductors placed in an external electric field [41], we propose to use a variant of the PSO algorithm, called the random drift particle swarm optimization (RDPSO), in order to achieve our goal of effectively estimating the parameters of complex biochemical dynamical systems. The motivation of the RDPSO algorithm is to improve the search ability of the PSO algorithm by fundamentally modifying the update equation of the particle's velocity, instead of by revising the algorithm based on the original equation so as to probably increase the complexity of the algorithmic implementation as well as its computational cost. It is different from the drift particle swarm optimization (DPSO) proposed by us in [42,43] in that it can make a better balance between the global search and the local search of the particle swarm.

The original and basic RDPSO version was recently introduced by us in [44], which was used for solving other problems in [45,46]. A novel variant of RDPSO algorithm is being proposed in this work to solve the parameter identification problem for two biochemical systems. The novel variant proposed here is different from the original one in that it employs an exponential distribution for sampling the velocity of the particles, whilst the original one used the Gaussian distribution.

The novel RDPSO variant is used for estimating the parameters of two benchmark models, one of which describes the thermal isomerization of α-pinene with 5 parameters [22,47], the other of which has a three-step pathway with 36 parameters [19]. The results of RDPSO and some other well-known global optimization algorithms are then compared and discussed. It should be noted that although this paper is focused on the parameter estimation for biochemical modelling, just as PSO and other EAs, the proposed RDPSO variant can be employed as a general-purpose tool for optimization problems in data miming tasks, such as clustering, classification, regression, and so forth, which widely exist in bioinformatics and computational biology [1,2,8,48,49].

## Methods

### Problem statement

The inverse problem of a nonlinear dynamic system involves finding proper parameters so as to minimize the cost function of the model with respect to an experimental data set, with some given differential equality constraints as well as other algebraic constraints. Such a data-driven regression problem can be approached with statistical techniques, using the given experimental data and the proposed models with unknown parameters. As stated by Moles et al. [19], the problem can be mathematically formulated as a nonlinear programming problem (NLP) with differential-algebraic constraints, whose goal is to find *θ *so as to minimize

(1) $$J=\overset{t_{f}}{\underset{0}{\int }}{\left(y_{\text{msd}}\left(t\right)-y\left(\theta ,t\right)\right)}^{T}W\left(t\right)\left(y_{\text{ msd}}\left(t\right)-y\left(\theta ,t\right)\right)dt$$

subject to

(2) $$f\left(\frac{d\text{x}}{dt},\text{x},\text{y},\theta ,\text{v},t\right)=0$$

(3) $$\text{x(}t_{0})={\text{x}}_{0}$$

(4) h(x,y,θ,t)=0

(5) g(x,y,θ,t)≤0

(6) $$\theta^{L}\leq \theta \leq \theta^{U}$$

where *J *is the cost function of the model, *θ *is a vector of model parameters to be estimated, $y_{\text{msd}}\left(t\right)$ is the experimental measure of a subset of the output state variables, y(θ,t) is the prediction of those outputs by the model, **x **is the differential state variables and **v **is a vector of other (usually time-invariant) parameters that are not to be estimated. In Equation (1), W(t) is the weighting (or scaling) matrix, and the equation can be discretized into a weighted least-square estimator. In Equation (2), *f *is the set of differential and algebraic equality constraints describing the system dynamics (i.e., the nonlinear process model). Equation (3) gives the initial value of x. In Equations (4) and (5), *h *and *g *are equality and inequality path and point constraints on system performance. In addition, *θ *is subject to upper and lower bounds, which are described by inequality constraints (6).

The above defined inverse problem is generally a multimodal (non-convex) optimization problem with multiple local optima due to the nonlinearity and constraints of the system dynamics. Even though many local and global optimization methods have been proposed to solve the problem as mentioned in Introduction, it is still challenging and very necessary to develop efficient optimization algorithms to deal with the parameter estimation problems, especially those for the dynamic systems with many parameters and many equations. Therefore this study focuses on the optimization approach for the inverse problem using the proposed variant of random drift particle swarm optimization (RDPSO) and other global optimization methods.

### Particle swarm optimization

The original PSO algorithm was introduced by Kennedy and Eberhart in [50]. The algorithm was inspired by the observed social behavior of bird flocks or fish schooling, and it roots its methodology both in evolutionary computing and artificial life. It shares many similarities with EAs, in that both the PSO and the EAs are initialized randomly with a population of candidate solutions and then update the population iteratively, in order to approximate the global optimal solution to the given problem. However, unlike EAs, PSO has no evolution operators such as crossover and mutation, but perform optimization tasks by updating the particles' position (potential solutions) according to a set of discrete differential equations. It was shown that the PSO algorithm has comparable and even better performance than GAs [51]

In the PSO with *m *particles, each particle *i *(1≤i≤m), representing a potential solution of the given problem in a *D*-dimensional space, has three vectors at the *k*^th ^iteration, namely, the current position $X_{i}^{k}=\left(X_{i,1}^{k},X_{i,2}^{k},\cdots \;,X_{i,D}^{k}\right)$, the velocity $V_{i}^{k}=\left(V_{i,1}^{k},V_{i,2}^{k},\cdots \;,V_{i,D}^{k}\right)$ and its personal best (*pbest*) position $P_{i}^{k}=\left(P_{i,1}^{k},P_{i,2}^{k},\cdots \;,P_{i,D}^{k}\right)$, which is defined as the position with the best objective function value found by the particle since initialization. A vector$G^{k}=\left(G_{1}^{k},G_{2}^{k},\cdots \;,G_{D}^{k}\right)$, called the global best (*gbest*) position, is used to record the position with the best objective function value found by the all the particles in the particle swarm since initialization. With the above specification, the update equations for each particle's velocity and current position are given by:

(7) $$V_{i,j}^{k+1}=w\cdot V_{i,j}^{k}+c_{1}r_{i,j}^{k}\left(P_{i,j}^{k}-X_{i,j}^{k}\right)+c_{2}R_{i,j}^{k}\left(G_{j}^{k}-X_{i,j}^{k}\right)$$

(8) $$X_{i,j}^{k+1}=X_{i,j}^{k}+V_{i,j}^{k+1}$$

fori=1,2,⋯m;j=1,2⋯,D, where $c_{1}$ and $c_{2}$ are known as acceleration coefficients and *w *is called the inertia weight, which can be adjusted to balance the exploration and exploitation ability of each particle [52]. Without loss of generality, we assume that the PSO is used to solve the following minimization problem:

(9) $$\begin{array}{ccc} Minimize & f\left(X\right) & ,s.t. \end{array}\;X\in S\subseteq R^{D}\;$$

where f(X)is an objective function (or fitness function) and *S *denotes the feasible space. Consequently, $P_{i}^{k}$ can be updated by:

(10) $$P_{i}^{k}=\{\begin{array}{c} \begin{array}{cc} \begin{array}{cc} X_{i}^{k} & \text{ if} \end{array} & f(X_{i}^{k})<f(P_{i}^{k-1}) \end{array}\\ \begin{array}{ccc} P_{i}^{k-1} & \text{if} & f(X_{i}^{k})\geq f(P_{i}^{k-1}) \end{array} \end{array}$$

and $G^{k}$ can be found by:

(11) $$G^{k}=P_{g}^{k},\text{ where }g=\underset{1\leq i\leq m}{\text{arg min}}\left[f\left(P_{i}^{k}\right)\right]$$

In Equation (7), $r_{i,j}^{k}$ and $R_{i,j}^{k}$ are the sequences of two different random numbers with uniform distribution on the interval (0, 1), namely, $r_{i,j}^{k},R_{i,j}^{k}\text{\textasciitilde{}}U\left(0,1\right)$. In order to prevent the particle from flying away out of the search scope, $V_{i,j}^{k}$ is restricted on the interval $\left[-V_{\text{max}},V_{\text{max}}\right]$, where $V_{\text{max}}$ is also a user-specified algorithmic parameter.

Many researchers have proposed different variants of PSO in order to improve the search performance of the algorithm and proved this through empirical simulation [3-7,52-56]

### The proposed random drift particle swarm optimization

In [57], it was demonstrated that if the acceleration coefficients are properly valued, each particle converges to its local attractor,$p_{i}^{k}=\left(p_{i,1}^{k},p_{i,2}^{k},\cdots p_{i,D}^{k}\right)$, so that the convergence of the whole particle swarm can be achieved. Each coordinate of $P_{i}^{k}$ is given by:

(12) $$p_{i,j}^{k}=\frac{c_{1}r_{i,j}^{k}P_{i,j}^{k}+c_{2}R_{i,j}^{k}G_{j}^{k}}{c_{1}r_{i,j}^{k}+c_{2}R_{i,j}^{k}},1\leq j\leq D$$

which can be restated as

(13) $$p_{i,j}^{k}=\phi_{i,j}^{k}P_{i,j}^{k}+\left(1-\phi_{i,j}^{k}\right),1\leq j\leq D$$

where

(14) $$\phi_{i,j}^{k}=\frac{c_{1}r_{i,j}^{k}}{c_{1}r_{i,j}^{k}+c_{2}R_{i,j}^{k}}$$

In the PSO algorithm, c_1 _and c_2 _are set to be equal, and thus $\phi_{i,j}^{k}$ is a random number with uniform distribution on the interval (0, 1), i.e. $\phi_{i,j}^{k}\sim U\left(0,1\right)$.

During the search process of the PSO, as particles' current position are converging to their own local attractor, their current positions, *pbest *positions, local attractors and the *gbest *positions are all converging to one single point. The directional movement of each particle *i *towards $P_{i}^{k}$ resembles the drift motion of an electron in metal conductors placed in an external electric field. According to the free electron model [37], the electron has not only drift motion caused by the external electric field, but also a thermal motion, which appears to be a random movement. The superposition of the drift thermal motions makes the electron careen towards the location of the minimum potential energy. Thus, if the position of an electron in the metal is regarded as a candidate solution and the potential energy function as the objective function to be minimized, the movement of the electron resembles the process finding the minimum solution of the minimization problem.

The above facts can lead to a novel variant of PSO if the particle in PSO is assumed to behave like an electron moving in a metal conductor in an external electric field. More specifically, it can be assumed that at the *k*th iteration, each particle *i *has drift motion towards $P_{i}^{k}$ as well as a thermal motion, with their velocities in each dimension *j *denoted as $V1_{i,j}^{k+1}$ and $V2_{i,j}^{k+1}$, respectively. As a result, the velocity of the particle is given by $V_{i,j}^{k+1}=V1_{i,j}^{k+1}+V2_{i,j}^{k+1}$. In the drift particle swarm optimization proposed in [42,43], we assume that $V1_{i,j}^{k+1}$ follows a Maxwell distribution, say a Gaussian probability distribution, and the $V2_{i,j}^{k+1}$ is given by the social part plus the cognitive part in Equation (7). This velocity update equation appears to add some effectiveness to the search performance of the particle swarm but has some shortcomings. Firstly, the Gaussian distribution has a thin tail so that it has less opportunity to generate outliers. As a result, the thermal motion of the particle has less randomness and cannot significantly improve the particle's global search ability. Secondly, although the update equation of $V2_{i,j}^{k+1}$:

(15) $$V2_{i,j}^{k+1}=c_{1}r_{i,j}^{k}\left(P_{i,j}^{k}-X_{i,j}^{k}\right)+c_{2}R_{i,j}^{k}\left(G_{j}^{k}-X_{i,j}^{k}\right)$$

can guarantee the particle to converge towards its local attractor, the two random scaling coefficients add randomness to its motion, which means that the particle's position is sampled at uniformly random positions within the hyper-rectangle around the *gbest *position and its personal best position. It is not able to enhance the particle's global search ability because of the finite scope of the hyper-rectangle, but it may weaken its local search ability, which is the responsibility of the directional motion. Therefore, the velocity of the particle, which is given by the sum of $V1_{i,j}^{k+1}$ and$V2_{i,j}^{k+1}$, may not be able to make a good balance between the global search and the local search of the particle. In the present study, we employ a new way of determining $V1_{i,j}^{k+1}$ and$V2_{i,j}^{k+1}$.

Here, we assume that the velocity of the thermal motion $V1_{i,j}^{k+1}$ follows a double exponential distribution, whose probability density function and probability distribution function are

(16) $$f_{V1_{i,j}^{k+1}}\left(v\right)=\frac{1}{\sigma_{i,j}^{k}}e^{\frac{-2|v|}{\sigma_{i,j}^{k}}}$$

and

(17) $$F_{V1_{i,j}^{k+1}}\left(v\right)=1-e^{\frac{-2|v|}{\sigma_{i,j}^{k}}}$$

respectively, where *v *represents the value of the random variable $V1_{i,j}^{k+1}$ and $\sigma_{i,j}^{k}$ is the standard deviation of the distribution. By employing a stochastic simulation method, we can express $V1_{i,j}^{k+1}$ as

(18) $$V1_{i,j}^{k+1}=\frac{\sigma_{i,j}^{k}}{2}\phi_{i,j}^{k}$$

(19) $$\phi_{i,j}^{k}=\{\begin{array}{c} \begin{array}{cc} +\ln\left(1/u_{i,j}^{k}\right) & \begin{array}{cc} if & s>0.5 \end{array} \end{array}\\ \begin{array}{ccc} -\ln\left(1/u_{i,j}^{k}\right) & if & s\leq 0.5 \end{array} \end{array},$$

where

where *s *and $u_{i,j}^{k}$ are two different random numbers uniformly distributed on the interval (0,1), i.e. $\begin{array}{cc} s, & u_{i,j}^{k} \end{array}\sim U\left(0,1\right)$. As for the value of $\sigma_{i,j}^{k}$, an adaptive strategy is adopted to determine $\sigma_{i,j}^{k}$ by $\sigma_{i,j}^{k}=2\alpha |C_{j}^{k}-X_{i,j}^{k}|$, where $C^{k}=\left(C_{1}^{k},C_{2}^{k},\cdots \;,C_{D}^{k}\right)$ is called the mean best (*mbest*) position, defined as the mean of the *pbest *positions of all the particles, i.e. $\begin{array}{cc} C_{j}^{k}=(1/m)\sum_{i=1}^{m}P_{i,j}^{k} & \left(1\leq j\leq D\right) \end{array}$.

The velocity of the drift motion $V2_{i,j}^{k+1}$ may have many possible forms. However, the following simple linear expression is adopted in this study:

(20) $$V2_{i,j}^{k+1}=\beta \left(p_{i,j}^{k}-X_{i,j}^{k}\right)$$

where $p_{i,j}^{k}$ is determined by

(21) $$p_{i,j}^{k}=\phi_{i,j}^{k}P_{i,j}^{k}+(1-\phi_{i,j}^{k}),\begin{array}{cc} \begin{array}{cc} & \phi_{i,j}^{k}\sim U(0,1), \end{array} & 1\leq j\leq D \end{array}$$

It can be immediately proven that if $V_{i,j}^{k+1}=V2_{i,j}^{k+1}$, when k→∞, $X_{i,j}^{k}\to p_{i,j}^{k}$. Therefore the expression of $V2_{i,j}^{k+1}$ in Equation (20) can indeed guarantee that the particle move directionally to $P_{i}^{k}$ as an overall result.

With the definitions of the thermal motion and the drift motion of the particle, we can obtain a novel set of update equations for the particle:

(22) $$V_{i,j}^{k+1}=\alpha |C_{j}^{k}-X_{i,j}^{k}|\phi_{i,j}^{k}+\beta \left(p_{i,j}^{k}-X_{i,j}^{k}\right)$$

(23) $$X_{i,j}^{k+1}=X_{i,j}^{k}+V_{i,j}^{k+1}$$

where *α *is called the thermal coefficient and *β *is called the drift coefficient. The PSO with Equations (22) and (23) is a novel variant of RDPSO, which employs a double exponential distribution instead of a Gaussian one. The procedure of this RDPSO variant is outlined below.

**Figure 1:**
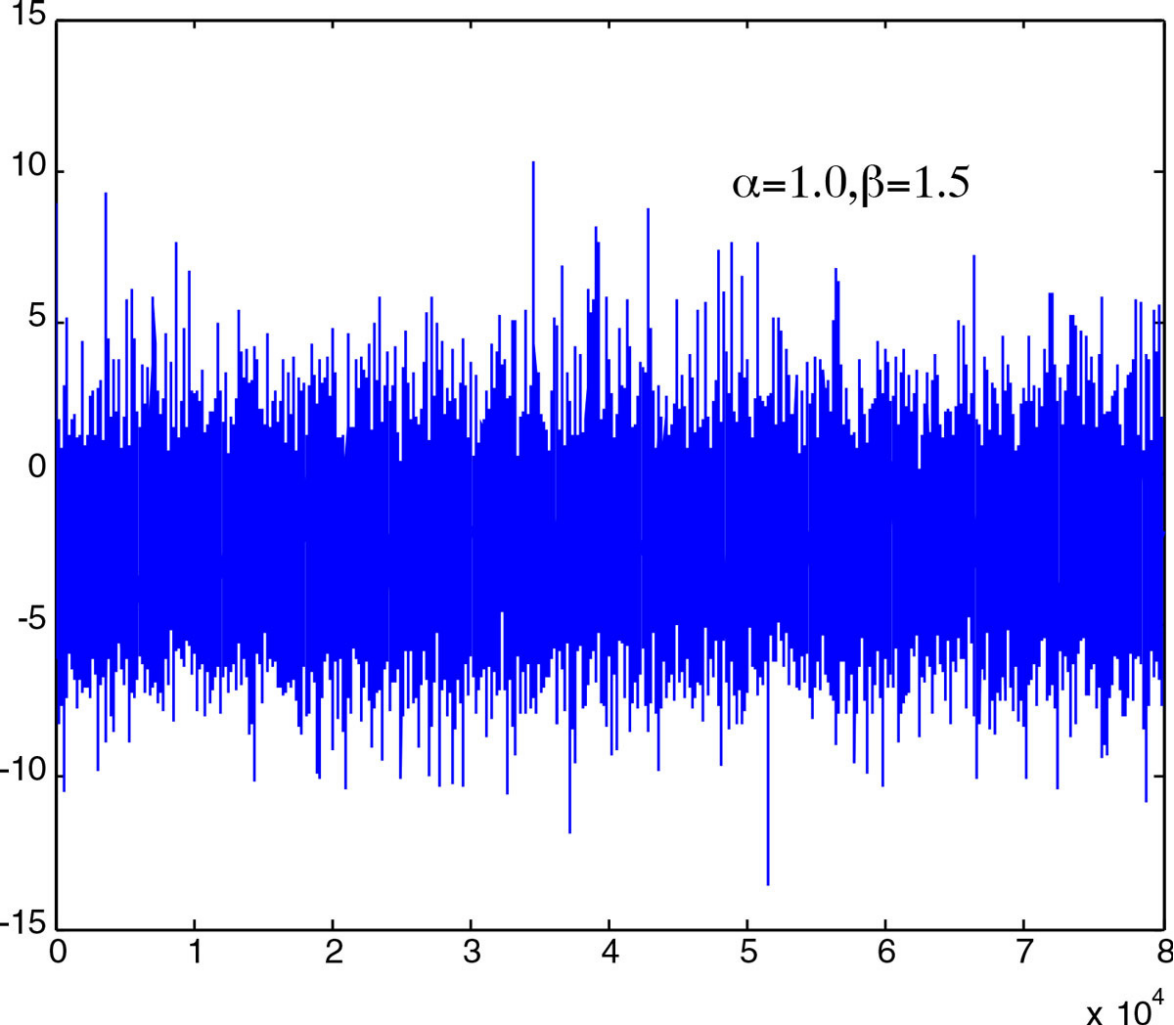
It is shown that, with this parameter setting, the particle's position is bounded.

**Figure 2:**
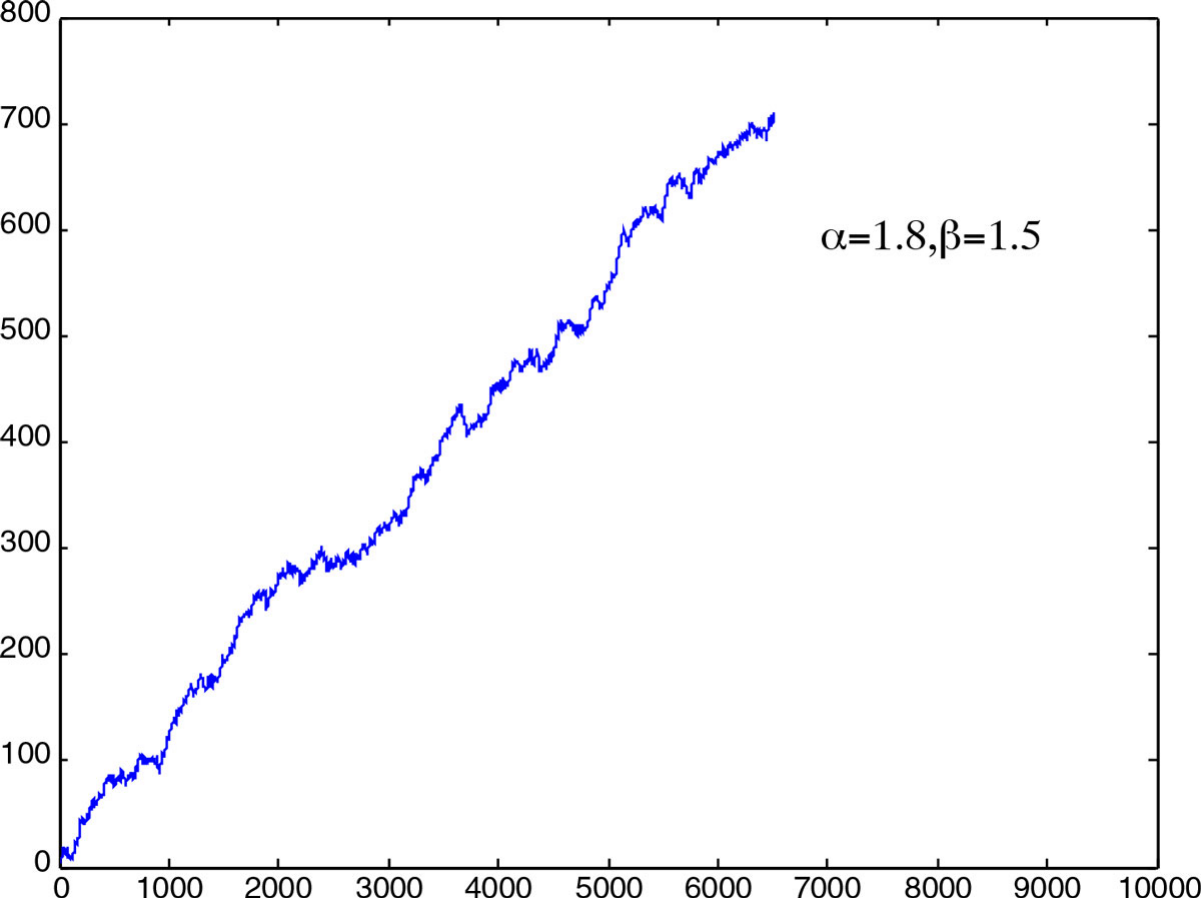
It is shown that, with this parameter setting, the particle's position diverges as the iteration number increases.

Step 0: Randomly initialize the current positions and the *pbest *position of all the particles;

Step 1: Set *k *= 0;

Step 2: While the termination condition is not met, do the following steps;

Step 3: Set *k*=*k*+1 and compute the *mbest *position $C^{k}$, which is the centroid of the *pbest *positions of all the particles at iteration *k*;

Step 4: From i=1, carry out the following steps;

Step 5: Evaluate the objective function value $f\left(X_{i}^{k}\right)$, and update $P_{i}^{k}$ and $G^{k}$ according to Equation (10) and Equation (11), respectively;

Step 6: Update the components of the velocity and current position of particle *i *in each dimension, respectively, according to Equations (21), (22) and (23);

Step 7: Set *i*=*i*+1, and return to Step 5 until i=m;

Step 8: Return to Step 2;

In the RDPSO algorithm, in addition to the population size *m*, *α *and *β *are two very important user-specified algorithmic parameters, which play the same roles as the inertia weight *w *and acceleration coefficients in the basic PSO algorithm. That is, the can be tuned to balance the exploration and exploitation ability of the particle. How to select the values of these parameters to prevent the particles from explosion is an open problem. Here, we performed the stochastic simulations for the one dimensional case, in which the local attractor was fixed at the origin and the *mbest *position was at *x *= 0.1. The results of two simulations are visualized in Figure 1 and Figure 2, in which the logarithmic value of the absolute of $X_{k}$ was recorded as ordinate, and the iteration number was the abscissa. Figure 1 shows that the particle's position was bounded when α=1 and β=1.5. However, when α=1.8 and β=1.5, the particle diverged to infinity. To obtain the sufficient and necessary condition for the particle to be bounded, we will focus our attention on a theoretical analysis in terms of probability measure in future.

Setting large values for *α *and *β *implies better global search ability of the algorithm, while setting small values means better local search. When the RDPSO is used for solving a problem, a good balance between the global search and the local search of the algorithm is crucial for the algorithmic performance. However, in order to find out how to tune the parameters to generate generally good algorithmic performance we need a large number of experiments on benchmark functions, which will be performed in our future tasks. Here, we recommend that when the RDPSO is used, *α *should be set to be no larger than 1.0 and *β *to be no larger than 1.5. More specifically, when the problem at hand is complex, the values of the two parameters should be set to be relatively large in order to make the particles search more globally, and on the other hand, when the problem is simple, relatively smaller values should be selected for the parameters, for the purpose of faster convergence speed of the algorithm. In the present study, the value of *α *and *β *were set to be 0.75 and 1.0, respectively.

In addition, the population size and the maximum number of iterations (MaxIter) can also affect the performance of a population-based technique. Just as for other PSO variants, it is suggested that the population size should be larger than 20 for the RDPSO as well. The value of the MaxIter depends on the complexity of the problem. Generally, a smaller MaxIter value is used for simple problems, while a larger one is used for complex problems.

Moreover, $V_{i,j}^{k}$ is also restricted within the interval $\left[-V_{\text{max}},V_{\text{max}}\right]$during the search process of the RDPSO algorithm, just as in the original PSO algorithm.

### The optimization methods compared

Besides the PSO and RDPSO algorithms, the Differential Evolution (DE), Scatter Search (SS) method and two versions of Evolution Strategies were also used to solve the selected inverse problems, for performance comparison purposes. The DE method, as presented by Storn and Price [58], is an evolutionary computing method, which has a faster convergence speed than GAs and can find the global optimal solution of a multidimensional and multimodal function effectively [58].

The SS method is also a population-based search techniques originally developed by Glover [59]. In [22], a novel meta-heuristic method, which is the combination of the original SS method with a local search technique, was proposed to solve inverse problems. It was shown that the local search operator can accelerate the convergence speed significantly. Thus, in our experiments, we used this novel SS method for performance comparison.

Evolutionary strategy (ES) is an important paradigm of EAs, which imitates the effects that genetics produces on the phenotype, rather than the genotype as in GAs [60]. The two canonical versions of ES we used in this study are denoted by (*μ*, *λ*)-ES and (*μ *+ *λ*)-ES, where *μ *denotes the number of parents and *λ *the number of offspring. In the (*μ*, *λ*)-ES, the parents are deterministically selected from offsprings (*μ *< *λ *must hold), while in (*μ *+ *λ*)-ES, the parents are selected from both the parents and offsprings.

In addition, the performances of the above mentioned algorithms, including the RDPSO, are also compared with those of the SRES method. The SRES is a version of (*μ*, *λ*)-ES that uses stochastic ranking to handle the constraints, by adjusting the balance between the objective function and the penalty function on the course of the search [19], [61].

### Case studies

Two case studies involving two benchmark systems were carried out. For each system, we performed two groups of numerical experiments, one with noise-free simulation data, and the other with noisy simulation data.

#### Case study 1

The goal of this case study is to estimate the five rate constants of the homogeneous biochemical reaction describing the thermal isomerization of α-pinene, which is an organic compound of the terpene class, one of two isomers of pinene [22], [23]. The mathematical model of this process is formulated with the following linear equations:

(24) $$\frac{dy_{1}}{dt}=-\left(p_{1}+p_{2}\right)y_{1}$$

(25) $$\frac{dy_{2}}{dt}=p_{1}y_{1}$$

(26) $$\frac{dy_{3}}{dt}=p_{2}y_{1}-\left(p_{3}+p_{4}\right)y_{3}+p_{5}y_{5}$$

(27) $$\frac{dy_{4}}{dt}=p_{3}y_{3}$$

(28) $$\frac{dy_{5}}{dt}=p_{4}y_{3}-p_{5}y_{5}$$

where $\left(p_{1},p_{2}\cdots \;,p_{5}\right)$ is the vector of unknown coefficients to be estimated, $y_{1}$, $y_{2}$, $y_{3}$, $y_{4}$ and $y_{5}$ denote the concentrations of the α-pinene, dipentene, alloocimen, β-pyronene and a dimer, respectively.

#### Case study 2

This case study involves the inverse problem to identify 36 kinetic parameters of a nonlinear biochemical dynamic model formed by the following 8 ordinary differential equations that describe the variation of the metabolite concentrates with time 1 [19].

(29) $$\frac{dG_{1}}{dt}=\frac{V_{1}}{1+{\left(\frac{P}{Ki_{1}}\right)}^{ni_{1}}+{\left(\frac{Ka_{1}}{S}\right)}^{na_{1}}}-k_{1}\cdot G_{1}$$

(30) $$\frac{dG_{2}}{dt}=\frac{V_{2}}{1+{\left(\frac{P}{Ki_{2}}\right)}^{ni_{2}}+{\left(\frac{Ka_{2}}{M_{1}}\right)}^{na_{2}}}-k_{2}\cdot G_{2}$$

(31) $$\frac{dG_{3}}{dt}=\frac{V_{3}}{1+{\left(\frac{P}{Ki_{3}}\right)}^{ni_{3}}+{\left(\frac{Ka_{3}}{M_{2}}\right)}^{na_{3}}}-k_{3}\cdot G_{3}$$

(32) $$\frac{dE_{1}}{dt}=\frac{V_{4}\cdot G_{1}}{K_{4}+G_{1}}-k_{4}\cdot E_{1}$$

(33) $$\frac{dE_{2}}{dt}=\frac{V_{5}\cdot G_{2}}{K_{5}+G_{2}}-k_{5}\cdot E_{2}$$

(34) $$\frac{dE_{3}}{dt}=\frac{V_{6}\cdot G_{3}}{K_{6}+G_{3}}-k_{6}\cdot E_{3}$$

(35) $$\begin{array}{c} \frac{dM_{1}}{dt}=\frac{kcat_{1}\cdot E_{1}\cdot \left(\frac{1}{Km_{1}}\right)\cdot \left(S-M_{1}\right)}{1+\frac{S}{Km_{1}}+\frac{M_{1}}{Km_{2}}}\\ \begin{array}{cc} & - \end{array}\frac{kcat_{2}\cdot E_{2}\cdot \left(\frac{1}{Km_{3}}\right)\cdot \left(M_{1}-M_{2}\right)}{1+\frac{M_{1}}{Km_{3}}+\frac{M_{2}}{Km_{4}}} \end{array}$$

(36) $$\begin{array}{c} \frac{dM_{2}}{dt}=\frac{kcat_{2}\cdot E_{2}\cdot \left(\frac{1}{Km_{3}}\right)\cdot \left(M_{1}-M_{2}\right)}{1+\frac{M_{1}}{Km_{3}}+\frac{M_{2}}{Km_{4}}}\\ \begin{array}{cc} & - \end{array}\frac{kcat_{3}\cdot E_{3}\cdot \left(\frac{1}{Km_{5}}\right)\cdot \left(M_{2}-P\right)}{1+\frac{M_{2}}{Km_{5}}+\frac{P}{Km_{6}}} \end{array}$$

where$M_{1}$, $M_{2}$, $E_{1}$, $E_{2}$, $E_{3}$, $G_{1}$, $G_{2}$ and $G_{3}$ are the state variables representing the concentrations of the species involved in different biochemical reactions, and *S *and *P *are controlling parameters which are kept fixed at the initial values for each experiment. The inverse problem is then reduced to the optimization problem that fits the remaining 36 parameters represented by$\theta =\left(\theta_{1},\theta_{2},\cdots \;,\theta_{36}\right)$.

### Objective functions

The objective function (or fitness function) for the inverse problem in either of the two case studies is the discretization of Equation (1), which is formulated as the weighted sum of squares of the differences between the experimental and the predicted values of the state variables:

(37) $$J=\overset{n}{\underset{i=1}{\sum }}\overset{l}{\underset{j=1}{\sum }}w_{ij}{\left\{{\left[y_{\text{pred}}\left(i\right)-y_{\text{exp}}\left(i\right)\right]}_{j}\right\}}^{2}$$

where *n *is the number of data for each experiment, *l *is the number of experiments, $y_{\text{exp}}$ is the vector of experimental values of the state variables, and $y_{\text{pred}}$ is the vector of the values of state variables predicted by the model with a given set of parameters. In Case Study 1, each $w_{ij}$ was set to be 1 [22], while in Case Study 2, $w_{ij}$ was set as $w_{ij}={\left\{1/\text{max}{\left[y_{\text{pred}}\left(i\right)\right]}_{j}\right\}}^{2}$, which was used to normalize the contributions of each term [19].

### Obtaining simulation data

In order to evaluate the performances of the global optimization methods in finding the solution of the inverse problems, we chose a set of parameters for each model, which are considered as the true or nominal values. For Case Study 1, the true values of the parameters are *p*_1 _= 5.93e-5, *p*_2 _= 2.96e-5, *p*_3 _= 2.05e-5, *p*_4 _= 27.5e-5 and *p*_5 _= 4.00e-5. For Case Study 2, the nominal values of the model parameters are shown in Table 1.

**Table 1:** Experiment generation (simulation) and the nominal value.

*P*	0.05	0.13572		0.36840	1.0
***S***	**0.1**	**0.46416**		**2.1544**	**10**

**Parameter**	**Element of decision variables vector**	**Nominal value**	**Parameter**	**Element of decision variables vector**	**Nominal value**

*V*_1_	*θ*_1_	1	*V*_4_	*θ*_19_	0.1

*Ki*_1_	*θ*_2_	1	*K*_4_	*θ*_20_	1

*ni*_1_	*θ*_3_	2	*k*_4_	*θ*_21_	0.1

*Ka*_1_	*θ*_4_	1	*V*_5_	*θ*_22_	0.1

*na*_1_	*θ*_5_	2	*K*_5_	*θ*_23_	1

*k*_1_	*θ*_6_	1	*k*_5_	*θ*_24_	0.1

*V*_2_	*θ*_7_	1	*V*_6_	*θ*_25_	0.1

*Ki*_2_	*θ*_8_	1	*K*_6_	*θ*_26_	1

*ni*_2_	*θ*_9_	2	*k*_6_	*θ*_27_	0.1
*Ki*_2_	*θ*_10_	1	*kcat*_1_	*θ*_28_	1

*na*_2_	*θ*_11_	2	*Km*_1_	*θ*_29_	1

*k*_2_	*θ*_12_	1	*Km*_2_	*θ*_30_	1

*V*_3_	*θ*_13_	1	*kcat*_2_	*θ*_31_	1

*Ki*_3_	*θ*_14_	1	*Km*_3_	*θ*_32_	1

*ni*_3_	*θ*_15_	2	*Km*_4_	*θ*_33_	1

*Ka*_3_	*θ*_16_	1	*Kcat*_3_	*θ*_34_	1

*na*_3_	*θ*_17_	2	*Km*_5_	*θ*_35_	1

*k*_3_	*θ*_18_	1	*Km*_6_	*θ*_36_	1

(P and S are initial concentrations of the pathway substrate and product, and were varied and combined to generate a total of 16 sets of pseudo-experimental (simulation) measurements)

The pseudo-experimental data (essentially the simulation data) in either case were generated by substituting the chosen parameters into the dynamic model and performing fourth order Runge-Kutta method on the corresponding differential equations. For Case Study 2, the pseudo-measurements of the concentrations of metabolites, proteins, and messenger RNA species were the results of 16 different pseudo-experiments, in which, with the given nominal values for the parameters, the initial concentrates of the pathway substrate *S *and product *P *were varied for each experiment (simulation) as shown in Table 1. These simulated data represent the exact experimental results devoid of measurement noise and they were used as noise-free data for the first group of numerical experiments in each case study. In order to test the optimization methods for noisy data, we added a white noise to each of the original noise-free data:

(38) $$z^{\prime }=z+\sigma \varepsilon$$

where *z *and $z^{\prime }$ represents the original noise-free data and the resulting noisy data, respectively, *ε *is an random number with standard normal distribution, namely, ε~N(0,1), and *σ *is the standard deviation of the white noise. In our case studies, *σ *was set to 0.01 for both systems.

### Initial problem solver used

During the search of each global optimization algorithm, each potential solution (i.e., a set of estimation values for the parameters) was substituted into the dynamic model. Then, the fourth order Runge-Kutta method was performed on the corresponding system of differential equations to generate a set of predicted values of the output state variables, from which the objective function value (or fitness value) of the potential solution could be evaluated according to Equation (37) with the obtained pseudo-experimental (simulation) data. This process is known as the solution to the forward problem, which was embedded in the iterations of the search during the solving of the inverse problem with the algorithm.

### Experimental settings

For the sake of performance comparison, all the tested global optimization methods except the SS method (i.e., PSO, RDPSO, DE, (*μ*, *λ*)-ES, and (*μ *+ *λ*)-ES) were programmed in C++ on a VC++6.0 platform in Windows XP environment, and implemented on Intel Pentium Dual-Core E5300 2.6GHz PCs, each with 2 MB cache and 4 GB main memory. The SS method was implemented in Matlab 7.0, on the same platform, for the purpose of calling the local solver SQP in Matlab during the search process. The software for SS for inverse problems can be found on http://www.iim.csic.es/~gingproc/ssmGO.html.

The configuration of the algorithm parameters including the population sizes are listed in Table 2. In Case Study 1, each optimization algorithm ran 20 times with each run executed for 500 iterations; that is, the maximum number of iterations (MaxIter) is 500. In Case Study 2, each algorithm also ran 20 times with each run executed for 2250000 function evaluations, which is the same as that for the DE in [19]. Since the population size of each algorithm was 100, the value of MaxIter was 22500 in Case Study 2. For the SS method, the initial population size was 100, and 10 individuals were selected to perform the iterative search after initialization. Other parameters were selected according to recommendations from the corresponding references and/or our preliminary runs. For all the algorithms tested on the inverse problems, the statistical values of *J *were figured out and the results with best values of *J *were selected, processed and visualized with Matlab 7.0.

**Table 2:** Configuration of search parameters in different algorithms (F is an algorithmic control parameter used to control the size of the perturbation in the mutation operator for DE, CR is the crossover constant in DE, varphi is a parameter determining the standard deviation of the mutation in the evolution strategy).

Algorithm	RDPSO	SS method	PSO	DE	(*μ*, *λ*)-ES	(*μ *+ *λ*)-ES
Search parameters	Population Size = 100 *α *= 0.75 *β *= 1.0	Initial Population Size = 100, 10 selected individual after initialization	Population Size = 100 *w *= 0.729 c1 = 1.49;c2 = 1.49	Population Size = 100 F = 0.5 CR = 0.55	*λ *= 100 *μ *= 10 varphi = 1	*λ *= 100 *μ *= 10 varphi = 1

## Results

For Case Study 1, the statistical values of *J *from 20 search runs with 500 iterations by each algorithm are listed in Table 3. The best value of *J *(*J *= 1.3740e-014) for the numerical experiments with noise-free simulation data was obtained by using our proposed RDPSO algorithm after running for 0.0348h (about 2 minutes). For the experiment with noisy data, the RDPSO generated the best J value (*J *= 0.2023) as well. The proposed algorithm also showed the best performance on average among all tested methods, as shown by the mean value of *J *over 20 runs. In this case study, the basic PSO algorithm showed good performance on low-dimensional inverse problems.

**Table 3:** *J *values and computational time for the global optimization methods in Case Study 1

Results for the Experiments (simulations) with Noise-free Data						
**Algorithms**	**RDPSO**	**SS method**	**(*μ*, *λ*)-ES**	**PSO**	**DE**	**(*μ *+ *λ*)-ES**

Best Value of *J*	1.3740e-14	0.3930	301.9941	3.4461e-005	258.892	339.4941

Mean Value of *J*	2.5845e-004	0.5703	2.8522e+06	0.0225	2.3197e+03	1.9103e+06

Standard Deviation of *J*	3.5978e-04	0.1348	2.2673e+06	0.0183	704.7776	2.2118e+06

CPU time(h)	0.0347	--	0.0419	0.0330	0.0353	0.0419

**Results for the Experiments (simulations) with Noisy Data**						

**Algorithms**	**RDPSO**	**SS method**	**(*μ*, *λ*)-ES**	**PSO**	**DE**	**(*μ *+ *λ*)-ES**

Best Value of *J*	0.2023	0.7195	388.9242	0.2028	1.0273e+003	52.4817

Mean Value of *J*	0.2026	0.8361	2.1952e+05	0.2083	2.5488e+003	8.2498e+05

Standard Deviation of *J*	4.2918e-04	0.0743	3.2308e+05	0.0126	5393.6832	1.6245e+06

CPU time(h)	0.0349	--	0.0423	0.0338	0.0351	0.0422

**Figure 3:**
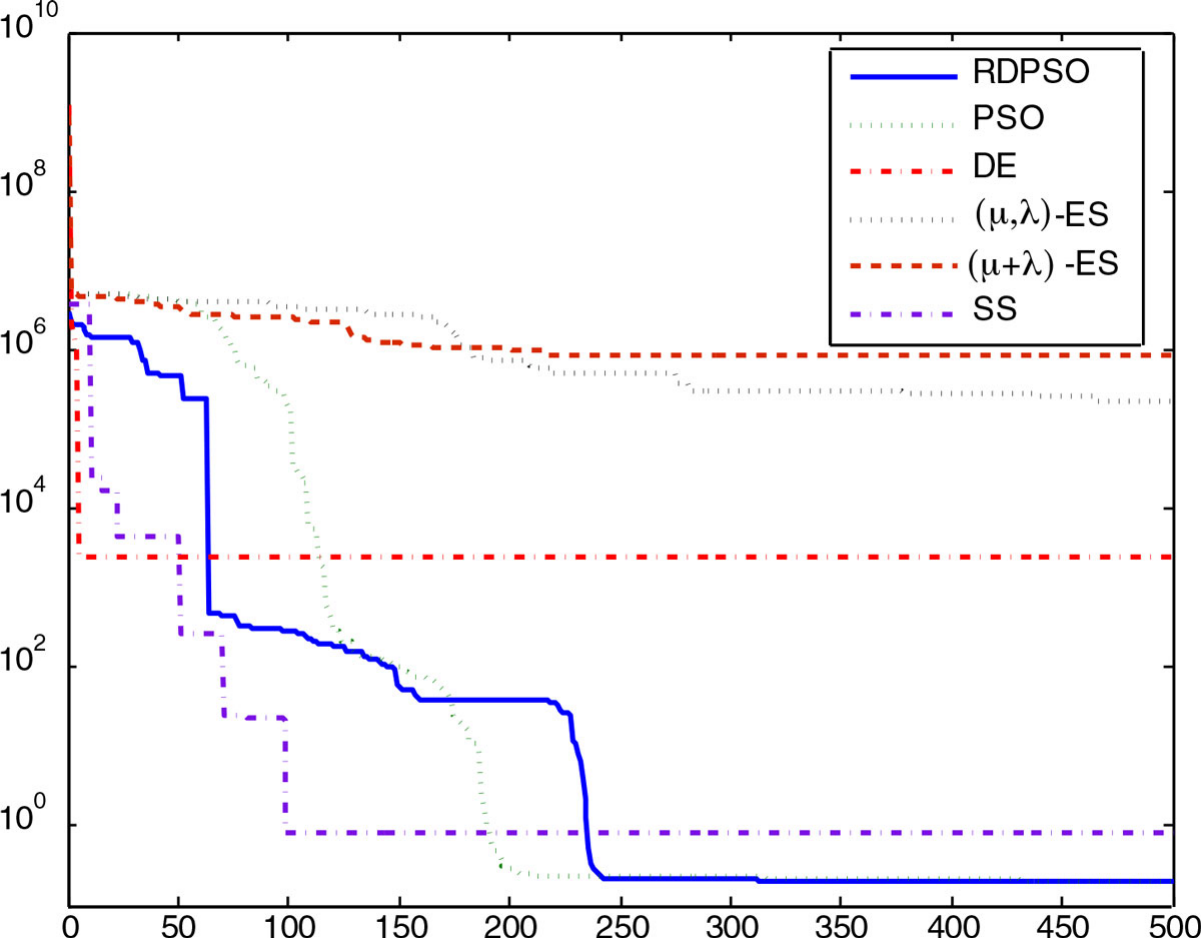
**The figure visualizes the convergence curves of objective function values of all the algorithms averaged over 20 runs in the numerical experiments with noisy data for Case Study 1**. It is shown that the RDPSO, PSO and SS methods had better convergence properties than other methods.

**Figure 4:**
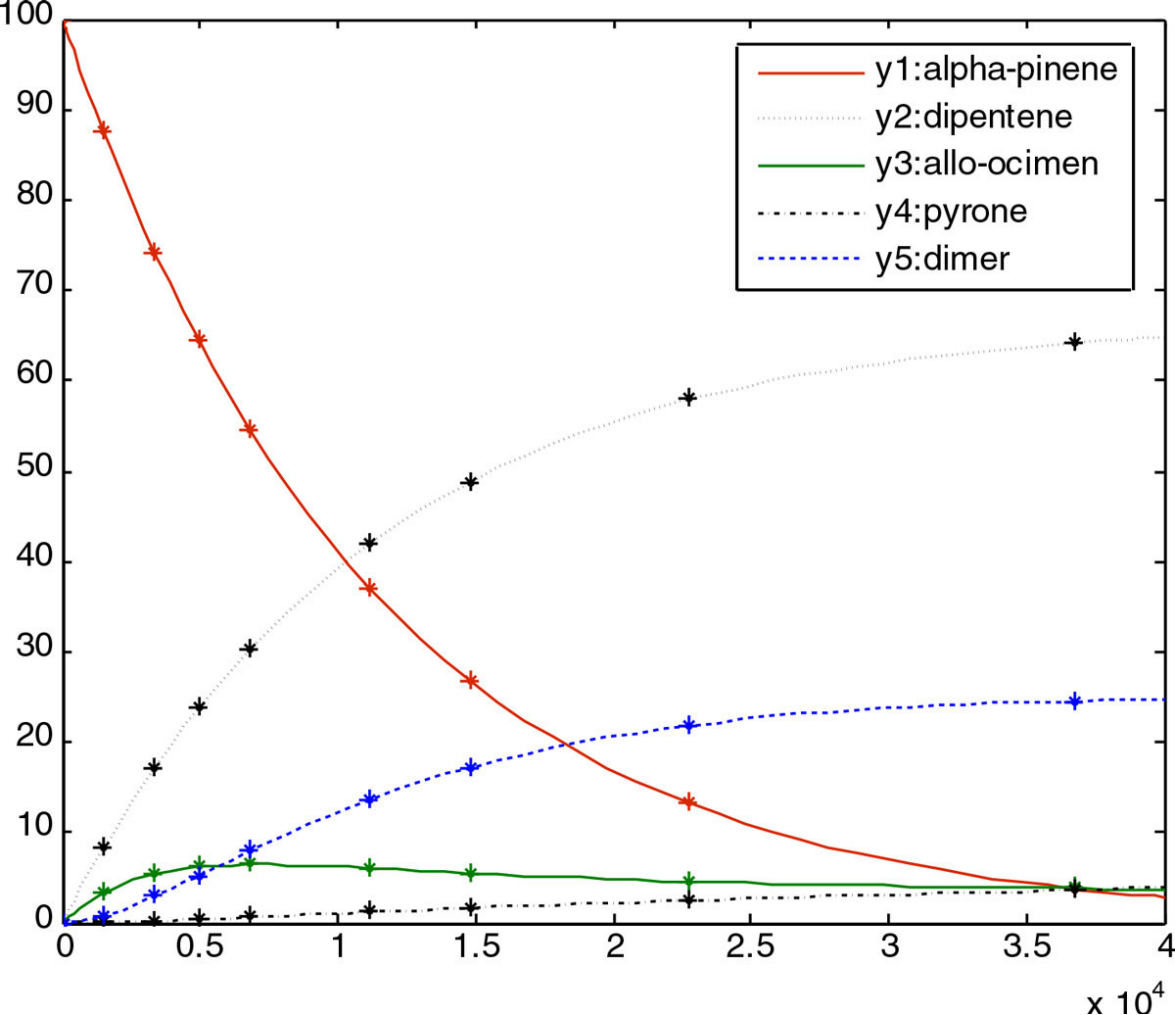
The figure shows the noise-free experimental data (marker) versus the predicted model (continuous line) for Case Study 1. It is shown that the predicted model obtained by RDPSO fits the experimental data well

**Figure 5:**
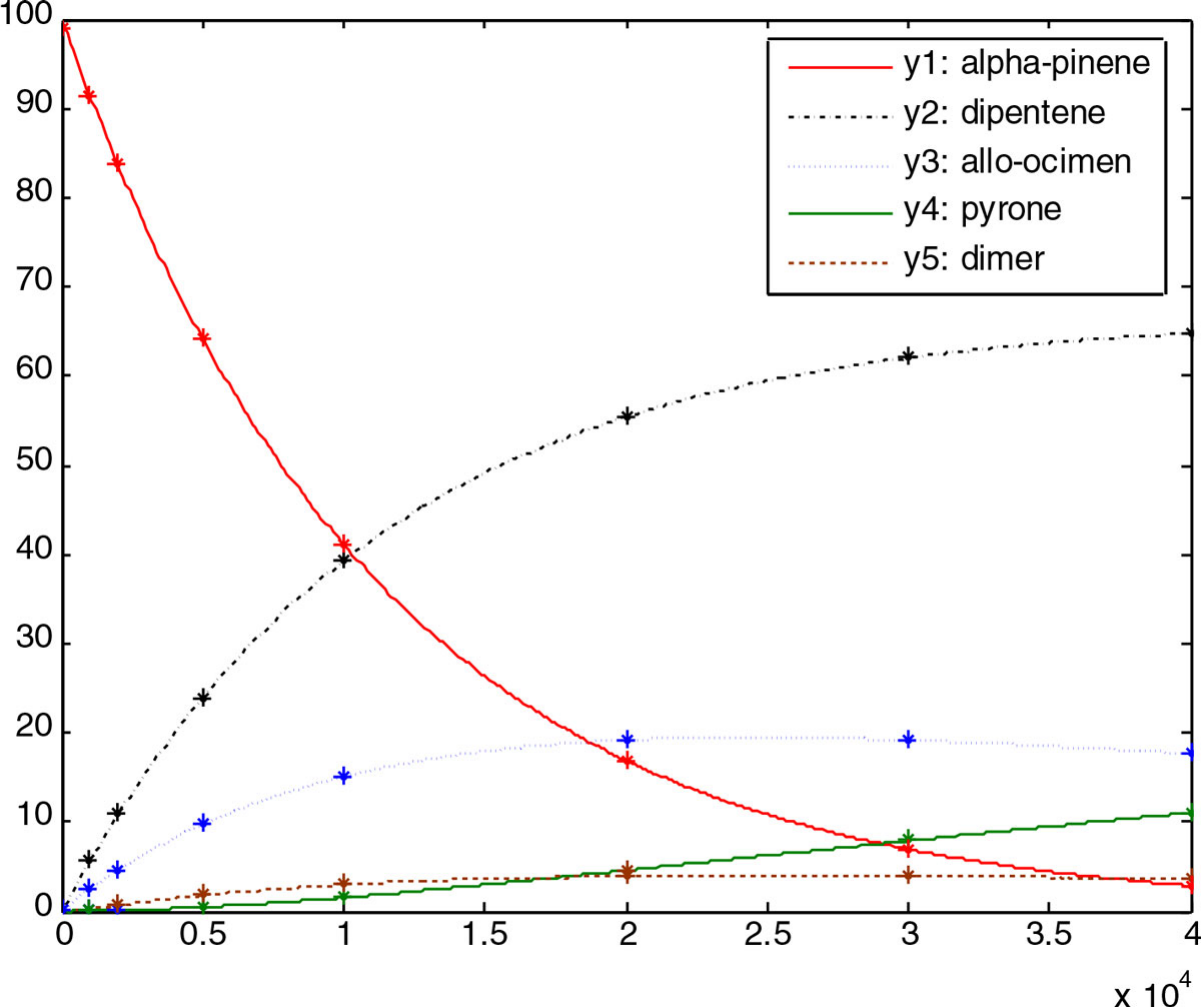
**The figure shows the noisy experimental data (marker) versus the predicted model (continuous line) for Case Study 1**. It is also shown that the predicted model obtained by RDPSO fits the experimental data well.

The convergence process of each tested algorithm averaged over 20 runs in the numerical experiment with noisy data in Case Study 1 is shown by the convergence curve in Figure 3, which is plotted in the log-log scale with objective function values versus the iteration number. Evidently, the SS method showed a better convergence property than other algorithms. The best solution vector corresponding to the best value of *J *(*J *= 1.3740e-014) obtained by the RDPSO in the numerical experiment with noise-free data was *p*_1 _= 5.930000e-005, *p*_2 _= 2.960000e-005, *p*_3 _= 2.750002e-004, *p*_4 _= 2.750002e-004, *p*_5 _= 4.000561e-005, extremely close to the real values of the parameters, and the best solution vector obtained by the RDPSO for the numerical experiment with noisy data was (when *J *= 0.2023) *p*_1 _= 5.928818e-005, *p*_2 _= 2.959918e-005, *p*_3 _= 1.712580e-005, *p*_4 _= 3.147252e-004, *p*_5 _= 1.537512e-003. Figure 4and Figure 5 show good fits between the experimental data (simulation data) and the predicted data, with the best obtained parameters in the experiments with both noise-free and noisy data.

For Case Study 2, the obtained values of *J *resulted from 20 runs with 22500 iterations by each algorithm are listed in Table 4. For the numerical experiments with noise-free data, the best result of *J *(*J *= 0.7358e-06) was obtained by using the SS method, which also had the better average performance than any other compared algorithm, as shown by the mean value of *J *over the 20 runs of the algorithm. The second best method was the RDPSO algorithm, which could converge to a value of *J *= 0.009124 and had a mean value of *J *= 0.178881 over 20 runs. Table 5 lists the estimated values of the model parameters corresponding to the best *J *value (*J=*0.009124) found by the RDPSO algorithm. The results also show that (*μ*, *λ*)-ES is the winner in this inverse problem compared to (*μ *+ *λ*)-ES, whose best and mean results of *J *were 0.123209 and 2.141820, respectively. The PSO and DE are two well-known efficient population-based optimization methods, which, however, could not arrive at the vicinity of the aforementioned solutions. When the experimental data (simulation data) was noisy, the SS method and RPSO obtained similar results for the best *J *value over 20 runs, but the former had a better average algorithmic performance. Table 6 shows the identified model parameters corresponding to the best *J *value (*J *= 0.2313) obtained by the RDPSO for the noisy data.

**Table 4:** *J *values and computational time of the global optimization methods for Case Study 2, including the results for the noise-free and noisy data.

Results for the Experiments with Noise-free Data						
**Algorithms**	**RDPSO**	**SS method**	**(*μ*, *λ*)-ES**	**PSO**	**DE**	**(*μ *+ *λ*)-ES**

Best Value of *J*	0.009124	7.1358e-07	0.022858	7.140163	10.168989	0.123209

Mean Value of *J*	0.178881	3.4.274e-06	0.736311	10.3859	17.701876	2.141820

Standard Deviation of *J*	0.252749	1.3649e-06	0.960729	3.1927	4.112377	1.692726

CPU time(h)	52.4	--	54.9	49.2	53.8	53.3

**Results for the Experiments with Noisy Data**						

**Algorithms**	**RDPSO**	**SS method**	**(*μ*, *λ*)-ES**	**PSO**	**DE**	**(*μ *+ *λ*)-ES**

Best Value of *J*	0.2313	0.2337	2.5957	7.7433	11.7900	5.1490

Mean Value of *J*	0.3459	0.3106	3.6029	11.2353	18.5928	10.8691

Standard Deviation of *J*	0.1268	0.1325	0.1730	3.2921	3.3616	3.7065

CPU time(h)	52.4	--	54.9	49.2	53.8	53.3

**Table 5:** Decision vector for the solution found by RDPSO in the experiment with noise-free data for Case Study 2.

Elements of best vector				
*θ*_1_-*θ*_4_	0.890001	0.996928	1.990488	1.000000

*θ*_5_-*θ*_8_	1.998655	0.885005	1.000085	1.000213

*θ*_9_-*θ*_12_	1.898600	0.999900	2.002120	1.010091

*θ*_13_-*θ*_16_	0.998763	1.005851	2.000021	0.995512

*θ*_17_-*θ*_20_	2.000150	0.996318	0.100025	1.000013

*θ*_21_-*θ*_24_	0.100211	0.099875	1.000361	0.100021

*θ*_25_-*θ*_28_	0.100004	1.000300	0.100008	1.000750

*θ*_29_-*θ*_32_	1.000321	0.987620	1.000041	1.000035

*θ*_33_-*θ*_36_	0.997855	0.998856	1.000000	1.000001

**Table 6:** Decision vector for the solution found by RDPSO in the experiment with noisy data for Case Study 2

Elements of best vector				
*θ*_1_-*θ*_4_	1.0128	0.9962	1.9923	1.0220

*θ*_5_-*θ*_8_	1.9698	1.0057	1.4290	0.7919

*θ*_9_-*θ*_12_	1.8388	1.6173	1.4905	0.9384

*θ*_13_-*θ*_16_	1.2683	1.0180	1.3832	0.9271

*θ*_17_-*θ*_20_	2.0248	1.3612	0.1242	1.4658

*θ*_21_-*θ*_24_	0.0956	0.1101	0.5970	0.1482

*θ*_25_-*θ*_28_	0.0979	1.0012	0.0977	0.9916

*θ*_29_-*θ*_32_	1.3351	1.9900	1.3957	1.9209

*θ*_33_-*θ*_36_	1.5504	1.3801	1.6637	1.1668

**Figure 6:**
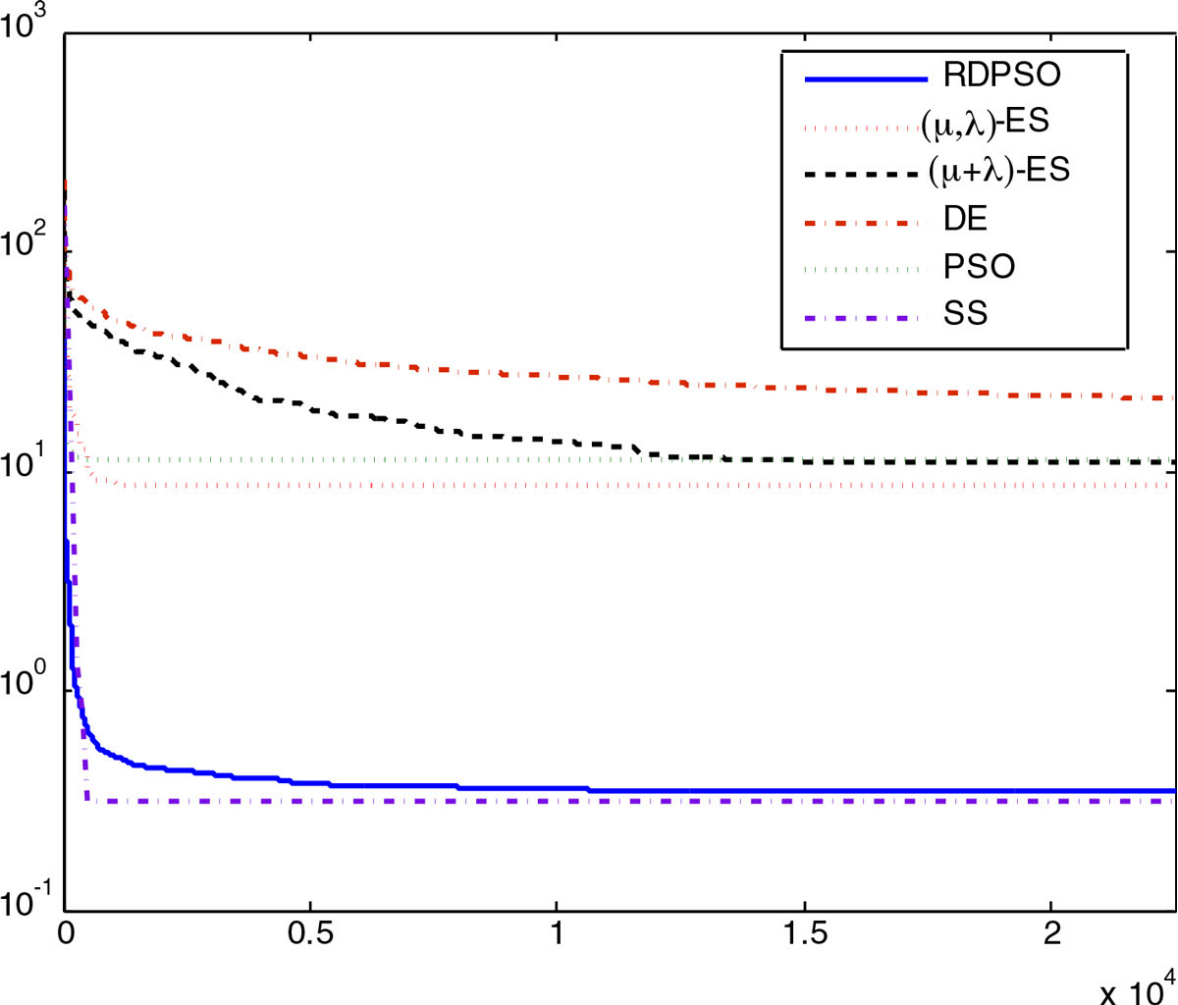
**The figure visualizes the convergence curves of objective function values of all the algorithms averaged over 20 runs of the numerical experiments with noisy data in Case Study 2**. It is shown that the SS method had the fastest convergence speed and the RDPSO had the second fastest one.

**Figure 7:**
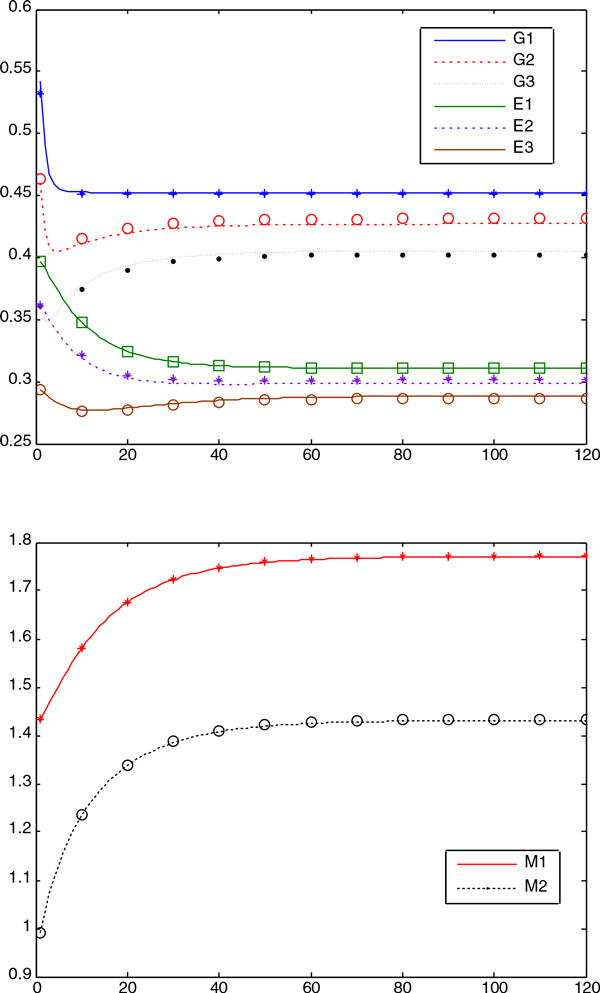
**The figure shows the predicted concentration provided by the RDPSO (continuous line) and noise-free experimental (marker) data in Case Study 2 when P = 1**.0, S = 2.1544. It is shown that the predicted concentration had good correlation with the experimental data.

We plotted in Figure 6 the convergence curve of each method averaged over 20 runs. It is shown that the SS method had a remarkably better convergence rate than others, probably due to its local solver that can enhance the local search ability of the algorithm significantly. Figures 7andFigure 8 show comparisons between the predicted data and the experimental (simulation) data for the decision vectors found by the RDPSO in both groups of numerical experiments (with the noise-free and noisy data, respectively). It can be observed that there is good correlation between the experimental and predicted data.

## Conclusions

In this paper, a variant of RDPSO algorithm was proposed and showed to be able to successfully solve two inverse problems associated with the thermal isomerization of α-pinene and a three-step pathway, respectively. The results indicate that the proposed RDPSO algorithm outperformed its PSO predecessors and some other competitors in the first problem, and also had the second best algorithmic performance among all the compared algorithms.

Like other stochastic optimization methods, a possible drawback of the RDPSO method is the computational effort required. This is mainly because most of the computational time was spent on solving the forward problem. One measure that can be taken is to incorporate the local search technique into the algorithm in order to accelerate its convergence speed. Another is to develop a parallelized RDPSO implementation to solve inverse problems on computer clusters to reduce the computational cost to a reasonable level. Our future tasks will focus on these two ways of improving the algorithmic effectiveness of the RDPSO algorithm.

## Availability and requirements

In the additional file 1 the source codes of five of the tested algorithms on the two benchmark systems are provided. It includes two file folds, one for benchmark system 1 and the other one for benchmark system 2. All the algorithms are programmed with C++ in Microsoft Visual C++ 6.0.

In additional file 2 the data files for the five algorithms used in the two case studies are provided. For each case, the data corresponding to the best results generated by 20 runs of each algorithm are provided in a .txt file.

## Authors' contributions

JS and VP designed the algorithm and drafted the manuscript. YC processed the experimental results. WF and XW revised the manuscript critically. All of the authors have read and approved the final manuscript.

## Competing interests

The authors declare that they have no competing interests.

**Figure 8:**
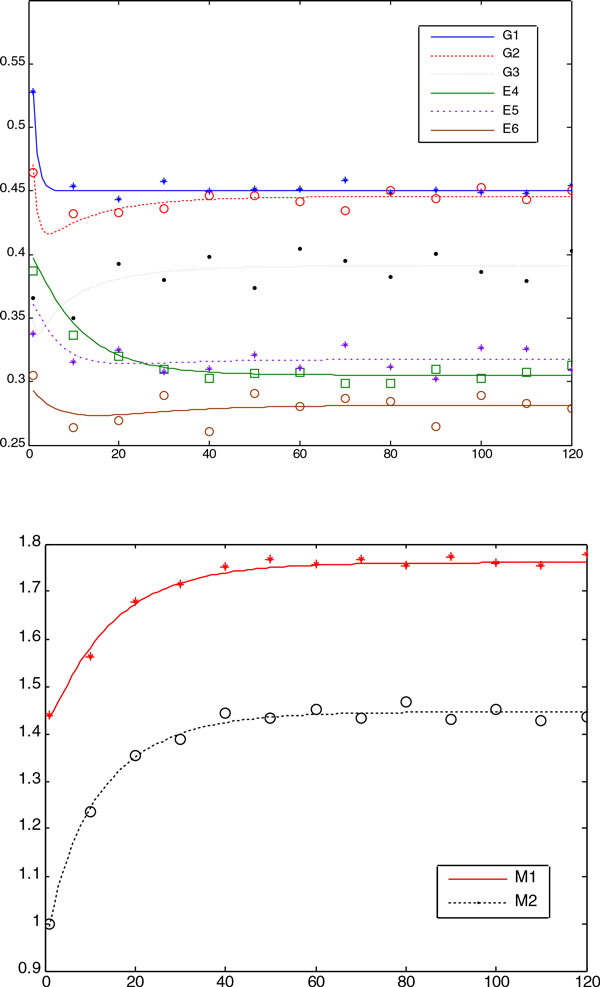
**The figure shows the predicted concentration provided by the RDPSO (continuous line) and noisy experimental (marker) data in Case Study 2 when P = 1**.0, S = 2.1544. It is shown that the predicted concentration had good correlation with the experimental data.

## Supplementary Material

Additional file 1 **Source. Code**This file includes the source code of all tested algorithms on the two benchmark problems, programmed in C++ on Microsoft Visual VC++ 6.0. All the source codes are compressed into a single .rar file. Click here for file

Additional file 2 **Data**. This file includes the data of the best solution out of 20 runs of each tested algorithm. Click here for file

## Declarations

Funding for the publication of this article comes from the Natural Science Foundation of China (NSFC) grant 61170119.

This article has been published as part of *BMC Bioinformatics *Volume 15 Supplement 6, 2014: Knowledge Discovery and Interactive Data Mining in Bioinformatics. The full contents of the supplement are available online at http://www.biomedcentral.com/bmcbioinformatics/supplements/15/S6.
