# Supplementary material for: Biochemical systems identification by a random drift particle swarm optimization approach
Source: BMC Bioinformatics. 2014 May 16;15(Suppl 6):S1. doi: 10.1186/1471-2105-15-s6-s1 (PMC4158603; doi:10.1186/1471-2105-15-s6-s1)
Supplement: Additional file 1 — Source. CodeThis file includes the source code of all tested algorithms on the two benchmark problems, programmed in C++ on Microsoft Visual VC++ 6.0. All the source codes are compressed into a single .rar file. [file 1471-2105-15-S6-S1-S1.zip › source-codes/thermal isomertization of alpha-pinene/RDPSO_ParameterEstimation_ThermalIsomerization/RDPSO_~1.PLG]

```
# Build Log


### --------------------Configuration: RDPSO_ParameterEstimation_ThermalIsomerization - Win32 Debug--------------------


### Command Lines

Creating temporary file "C:\DOCUME~1\nbstar\LOCALS~1\Temp\RSP23A.tmp" with contents
[
kernel32.lib user32.lib gdi32.lib winspool.lib comdlg32.lib advapi32.lib shell32.lib ole32.lib oleaut32.lib uuid.lib odbc32.lib odbccp32.lib /nologo /subsystem:console /incremental:yes /pdb:"Debug/RDPSO_ParameterEstimation_ThermalIsomerization.pdb" /debug /machine:I386 /out:"Debug/RDPSO_ParameterEstimation_ThermalIsomerization.exe" /pdbtype:sept 
".\Debug\RDPSO_ParameterEstimation_ThermalIsomerization.obj"
]
Creating command line "link.exe @C:\DOCUME~1\nbstar\LOCALS~1\Temp\RSP23A.tmp"

### Output Window

Linking...

### Results

RDPSO_ParameterEstimation_ThermalIsomerization.exe - 0 error(s), 0 warning(s)
```
